# Digital Parent–Child Intervention on Children’s Exercise Behavior and Psychological Development—A Randomized Controlled Trial Based on Family Perspective

**DOI:** 10.3390/bs16020282

**Published:** 2026-02-15

**Authors:** Yijuan Lu, Shengsheng Li, Zhen Xie, Feijun Meng, Rui Feng, Yijia Zhang, Wu Zhou, Yue Gao

**Affiliations:** 1 School of Physical Education, Hangzhou Normal University, Hangzhou 311121, China; luyijuan@hznu.edu.cn (Y.L.);; 2 Hangzhou Dongrun Foreign Language School, Hangzhou 311215, China

**Keywords:** physical activity volume, physical exercise attitude, mental health, intervention

## Abstract

**Objective:** From a family perspective, this study aimed to examine the effects of a 12-week digital platform-based parent–child exercise intervention on children’s behavioral level (physical activity), psychological level (physical exercise attitude), and mental health. **Methods:** This study included 218 students aged 10–11 years who underwent a 12-week standardized parent–child exercise intervention. The intervention group completed two structured parent–child tasks per week through a digital platform (Ding Talk App) while maintaining regular physical education classes; the control group only maintained their regular physical education classes. Assessments were conducted using the Physical Activity Rating Scale, Exercise Attitude Scale, and mental health scales (The Spence Children’s Anxiety Scale (SCAS) and The Patient Health Questionnaire-9 (PHQ-9)) at four stages: pre-intervention (T1), mid-intervention (T2), post-intervention (T3), and a 2-month follow-up period (T4). The intervention effects and effect sizes (ηp^2^) were examined using Repeated Measures Analysis of Variance. **Results:** At the behavioral level, a significant group × time interaction was found for physical activity volume (F = 17.651, *p* = 0.04, ηp^2^ = 0.138), indicating the presence of a moderate effect. At the psychological level, significant interactions were observed across exercise attitude dimensions (behavioral attitude: F = 3.699, *p* = 0.002, ηp^2^ = 0.033; perceived behavioral control: F = 4.189, *p* = 0.006, ηp^2^ = 0.037; subjective norm: F = 4.616, *p* < 0.001, ηp^2^ = 0.040) and mental health measures (depression: F = 4.009, *p* = 0.026, ηp^2^ = 0.044; anxiety: F = 3.1, *p* = 0.016, ηp^2^ = 0.020), though no significant effect was found for behavioral intention (F = 1.346, *p* = 0.259, ηp^2^ = 0.012), with all significant effects being relatively weak. **Conclusions:** The home–school collaborative, digital platform-based parent–child exercise intervention demonstrated positive effects on children’s physical activity volume, exercise attitudes, and mental health, offering a feasible and promising strategy to support more integrated child health promotion initiatives.

## 1. Introduction

Childhood is a critical stage for the development of exercise habits, the shaping of attitudes towards physical exercise, and the cultivation of psychological resilience (Tong, 2022). The earlier interventions are implemented during this stage, the higher the return on investment for long-term individual development (Heckman, 2006). However, practical problems exist in the field of child health promotion across three dimensions: the child, the family, and the school.

Insufficient physical activity among children has become a global public health issue. Relevant reports from the World Health Organization indicate that the rate of insufficient physical activity among Chinese children and adolescents was as high as 84.3% in 2016 (Guthold et al., 2020), characterized by “prolonged sedentary behavior time and declining initiative in physical activity participation” (Mikalsen et al., 2024). This phenomenon is closely related to the lack of guidance for children’s physical activity behaviors. As children’s autonomous ability is not yet mature, the generation and development of their physical activity behaviors depend on guidance from adults such as teachers and parents (B. Liu & Rao, 2021). Physical exercise attitude, as a comprehensive manifestation of students’ cognitive evaluation, affective evaluation, and behavioral intention towards physical education learning and body exercise, is the core link connecting external guidance and actual exercise behavior (Mao, 2003). This association can be explained based on the Theory of Planned Behavior (TPB) proposed by Ajzen (1991). This theory states that behavioral intention is the direct antecedent of actual behavior, and the formation of behavioral intention is primarily directly regulated by three cognitive variables: attitude toward the behavior, subjective norm, and perceived behavioral control (Lu et al., 2022). Specifically, in the domain of children’s exercise, attitude can predict intention and behavior. When children form positive cognitions and affective tendencies towards exercise as being fun and beneficial, it first fosters the behavioral intention of “wanting to exercise.” The expectations of important groups like family and school regarding children’s exercise behavior (subjective norm) and children’s judgment of their own exercise ability (perceived behavioral control) further strengthen this intention, ultimately influencing the occurrence and persistence of actual exercise behavior. Research has confirmed that positive exercise attitudes, supportive subjective norms, and stronger perceived behavioral control can significantly enhance children’s intention to participate in exercise and their actual exercise volume (L. Wang & Zheng, 2020).

Furthermore, children’s mental health issues cannot be ignored. Survey data shows that among children and adolescents in China, the average score for depressive symptoms is 4.63 ± 5.12. Among them, 14.8% exhibit varying degrees of depressive manifestations (including “mild depression risk” and “severe depression risk”). Specifically, 4.0% belong to the severe depression risk group, and 10.8% are in the mild depression risk group (Fu, 2023). Although exercise intervention has been proven to be a “natural prescription” for improving children’s psychological state, with particularly prominent effects in alleviating depressive and anxiety symptoms and enhancing executive functions (Zhu et al., 2025), existing research mostly focuses on the direct improvement of psychological indicators by exercise dosage (Mammen & Faulkner, 2013). It fails to fully explore the potential of exercise forms combining parent–child interaction in psychological healing and lacks sustainable intervention plans suitable for family settings.

Parents, as the primary influencing agents in the process of child development, significantly promote children’s development across physiological, psychological (Gong et al., 2024), and social adaptation (Bu & Li, 2024) dimensions through participation in parent–child interactive activities. Parent–child exercise, as a specific form of physical activity, provides a stable and accessible environment for children’s participation in physical activity through parents’ continuous companionship and behavioral modeling. This high-frequency opportunity for joint exercise can directly increase children’s exercise duration and intensity, thereby significantly enhancing their overall physical activity volume (Rebold et al., 2016). Meanwhile, positive feedback and targeted encouragement conveyed by parents during the interaction (Määttä et al., 2014) can deepen children’s understanding of the value of physical activity, enhance their emotional experience, and positively shape their attitudes toward exercise and participation intentions (L. Wang & Zheng, 2020). Therefore, parent–child exercise not only serves as a significant vehicle for enhancing children’s physical and psychological well-being but also represents a cost-effective and feasible pathway for promoting the integrated development of family and national health initiatives (J. Zhao & He, 2023).

However, the promotion of parent–child exercise currently faces dual challenges in practice. At the family level, intervention methods remain relatively singular and rigid, primarily relying on approaches such as knowledge-based lectures for parents, assigned family exercise homework, and requirements for parental supervision (Z. Li et al., 2023). Traditional offline models (e.g., verbal instructions or spreadsheet-based tracking) often struggle to effectively monitor exercise progress or provide timely feedback due to a lack of supervision and insufficient formative evaluation from teachers during implementation (Luo & Wang, 2024). At the school level, they have significant advantages in educational resources and professional guidance but have shortcomings in their actual effectiveness. First, school physical education generally suffers from “classroom limitations,” lacking systematic guidance for the extension of physical activities into the family setting. This leads to difficulties in effectively maintaining and deepening the motor skills and habits children learn at school within the home environment (C. Zhang et al., 2022). Second, traditional school behavior education focuses on rule compliance and discipline maintenance, paying insufficient attention to deeper psychological elements such as the formation of children’s exercise-related attitudes and the cultivation of intentions (Yang & He, 2024). This results in the failure to effectively stimulate children’s intrinsic motivation to participate in physical activities, making it difficult to form autonomous participation awareness and a lifelong exercise attitude (Yang & He, 2024). Third, the school’s psychological education, which relies on thematic courses and group counseling, is also disconnected from the family physical activity scene. This disconnection means psychological education lacks the support of real-life scenarios, making it difficult to translate into children’s daily emotion regulation ability and social interaction skills. Although 80% of schools recognize the importance of family involvement, less than 35% provide parents with specific parent–child exercise guidance plans (Xiao & Li, 2020). This gap in home–school cooperation both limits the extended effectiveness of school education and weakens the professional support for family education.

This disconnection between family and school further reflects the current situation in the field of child health promotion, characterized by “fragmented domains and insufficient collaboration.” Exercise interventions focus on skill enhancement (Dong et al., 2021; P. Zhao, 2013), behavioral interventions are confined to classroom guidance (Ma, 2020), and psychological interventions are limited by insufficient professional resources (J. Li et al., 2023). There is a lack of intervention programs that simultaneously cover physical activity, exercise attitude, and mental health, and utilize digital means for integrated management and implementation. Against this background, the parent–child exercise model integrated with digital technology offers new possibilities for achieving manageable, feedback-enabled interactive exercise interventions in the family setting. This model can not only directly increase children’s opportunities for exercise participation but also promote the synergistic improvement of their behavioral tendencies and psychological state through structured, gamified parent–child interactions (Y. Liu et al., 2025; K. Wang et al., 2011). Unlike traditional parent–child exercise formats that primarily rely on one-way instruction or supervision, this study introduces a digital platform that enables structured guidance, process-based tracking, and real-time feedback for parent–child interactions. In contrast to current intervention practices characterized by a disconnect between families and schools, this model adopts the home as the routine setting for implementation and leverages the digital platform to achieve effective coordination and closed-loop management between families and schools throughout the children’s exercise process.

Based on the above, this study designs a 12-week randomized controlled trial (RCT) of a digital parent–child exercise intervention, aiming to promote positive behavioral and cognitive changes by constructing a supportive family microenvironment, and thereby improve emotional states. Specifically, the study hypothesizes that the intervention exerts its effects through the following chain mechanisms: First, structured and gamified parent–child tasks directly increase children’s physical activity volume (behavioral dimension). Second, during such interactions, parents’ participation, encouragement, and role-modeling help children form more positive cognitive evaluations of exercise (behavioral attitude), perceive stronger social support (subjective norm), and enhance their confidence in their own ability to complete exercise (perceived behavioral control), thus jointly shaping their positive exercise attitudes (psycho-cognitive dimension).

These positive changes at the behavioral and cognitive levels are expected to ultimately alleviate anxiety and depressive symptoms through multiple pathways:

(a) Physical activity itself can directly regulate the neuroendocrine system, such as promoting the release of neurotransmitters like endorphins, which generates an immediate mood-enhancing effect;
(b) The emotional support and companionship derived from parent–child interaction can directly strengthen children’s psychological security and sense of belonging, and buffer stress;
(c) Improved exercise competence and the sense of accomplishment gained from task completion will enhance children’s self-efficacy, which is a key psychological resource against helplessness and anxiety;
(d) Regular parent–child activities also help improve sleep quality, which is closely associated with emotional regulation.

Therefore, the purpose of this study is not only to examine the independent effects of the intervention on behavior, attitude, and mental health, but also to provide preliminary empirical support for the theoretical path of “family environment remodeling → increased behavioral participation → formation of positive cognition → improvement of emotional health”. Based on this, the following specific hypotheses were proposed for this study:

**H1:**  *Compared to the control group, children in the intervention group would demonstrate a significantly greater increase in physical activity volume during the 12-week intervention period and the follow-up stage.*

**H2:**  *Compared to the control group, children in the intervention group would show more positive improvement trends across various dimensions of exercise attitude (behavioral attitude, subjective norm, and perceived behavioral control); however, changes in their behavioral intention might not be significant.*

**H3:**  *Compared to the control group, children in the intervention group would exhibit a more pronounced reduction in anxiety and depression levels.*

**H4:**  *The increase in physical activity volume and the formation of positive exercise attitudes would play a mediating role in the process by which the parent–child exercise intervention improves children’s anxiety and depression symptoms.*

## 2. Materials and Methods

### 2.1. Trial Design

To evaluate the effects of the parent–child exercise intervention on children’s physical activity levels, physical exercise attitudes, and mental health, this study employed a parallel-design randomized controlled trial (RCT) conducted at Hangzhou Dongrun Foreign Language School. The study protocol was approved by the Ethics Review Committee of Hangzhou Normal University (Approval No.: 20240901) and adhered to the ethical principles of the Declaration of Helsinki throughout. Prior to the study, parent-informed consent forms were distributed to students in the target classes through official school channels. After obtaining signed parental consent, age-appropriate explanations were provided to the students on the trial commencement day, and their voluntary assent was obtained before formal inclusion in the study. All participants were informed that they could voluntarily withdraw at any time during the study without providing a reason. This study utilized a class-level cluster randomization scheme for group allocation. Eligible students were randomly assigned to either the intervention group (3 classes) or the control group (2 classes).

### 2.2. Masking

Given that this study involves behavioral interventions and psychological measurements, it was not feasible to implement masking for the participants or the teachers delivering the intervention. To control for potential bias, the following measures were taken:

First, assessor masking was employed. All research assistants responsible for collecting outcome indicators (including data on physical activity volume, physical exercise attitudes, and mental health) were kept unaware of the participants’ group assignments. Throughout the entire data collection process, they did not communicate with the intervention implementation team regarding group allocation, thereby ensuring the objectivity of the obtained results.

Furthermore, data analysis masking was utilized. During the data analysis phase, the statisticians responsible for the analysis were not exposed to any information that could reveal group membership. All data were processed in coded form. The masking was only lifted after the completion of all primary statistical analyses, at which point results were interpreted and groups were labeled.

### 2.3. Participants

**Figure 1:**
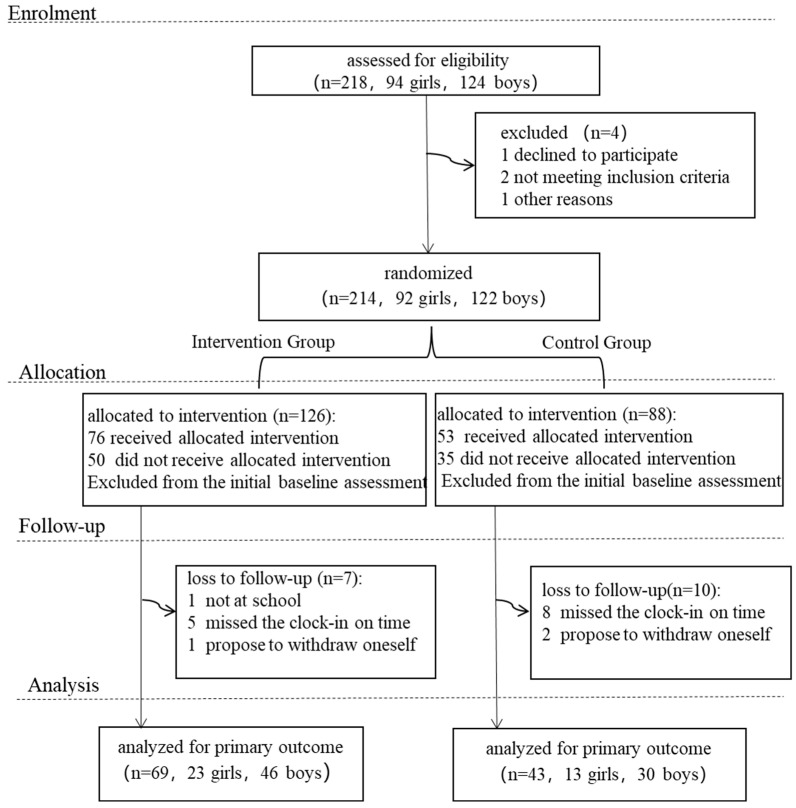
Participant Flow Diagram.

The sample size was calculated based on the anticipated effect size, significance level (α = 0.05), power (1 − β = 0.80), and the variability of the outcome measures. This study anticipated that the intervention would yield a medium effect size (Cohen’s d = 0.5), with the significance level set at 0.05 and power at 0.80. Accounting for a potential dropout rate (estimated at 50%), the sample size was calculated to ensure the statistical significance and robustness of the study findings. For the primary outcome measures, such as physical exercise attitude and mental health status, the calculation indicated a requirement of at least 40 participants per group. Consequently, this study recruited participants through schools. A total of 218 children were assessed for eligibility. After screening, individuals with severe physical illnesses or mental health conditions that could affect their participation in normal physical activities were excluded. Ultimately, 214 subjects met the study inclusion criteria. All participants were between 10 and 11 years old, were assessed to have the ability to understand and participate in the study activities, and their parents had provided informed consent. All enrolled subjects completed baseline data collection before the formal intervention began. During the 12-week intervention and subsequent follow-up period, some participants withdrew due to personal reasons, loss to follow-up, or failure to complete all assessments. The retention of participants in each group at different stages is detailed in the study participant flow diagram (Figure 1).

### 2.4. Intervention

#### 2.4.1. Rationale for Intervention Implementation

The development of this intervention program was primarily based on the following three considerations:

Firstly, it was based on mechanisms derived from Social Cognitive Theory. Existing research indicates that parent–child exercise has a significantly stronger negative predictive effect on children’s anxiety (β = −0.138) and depression (β = −0.145) compared to individual exercise alone (S. S. Li et al., 2025). This supports the concept of triadic reciprocal determinism (“environment-behavior-individual”) within Social Cognitive Theory. As a specific practice embedding family environmental factors into physical activity behavior, parent–child exercise can more effectively promote children’s positive emotional experiences and mental health through mechanisms such as parental modeling, observational learning, and emotional support. Therefore, this program is designed with parent–child interaction as the core component, aiming to systematically activate this theoretical mechanism.

Secondly, it aimed to address challenges to practical feasibility. Preliminary survey data also revealed that up to 46.7% of barriers to parent–child exercise stem from parental factors (e.g., work pressure, time allocation difficulties, and insufficient awareness of the psychological benefits of exercise) (S. S. Li et al., 2025). To overcome these practical barriers, this program adopted a hybrid intervention model of “regular physical education class + structured family parent–child tasks.” By pushing refined self-developed guidance materials and videos and utilizing the Ding Talk app check-in system, it aimed to lower the participation threshold for parents in terms of time and professional skills, thereby enhancing the intervention’s feasibility and family compliance.

Finally, it adhered to the principles of children’s physical and mental development. The program design followed scientific exercise principles, aiming to synchronously promote the coordinated development of children’s neuro-muscular and cardiorespiratory systems through quality training, avoiding the risk of developmental imbalance that might result from single-quality training. Thus, while improving mental health, it also consolidates the foundation of their physical health.

#### 2.4.2. Intervention Content

**Figure 2:**
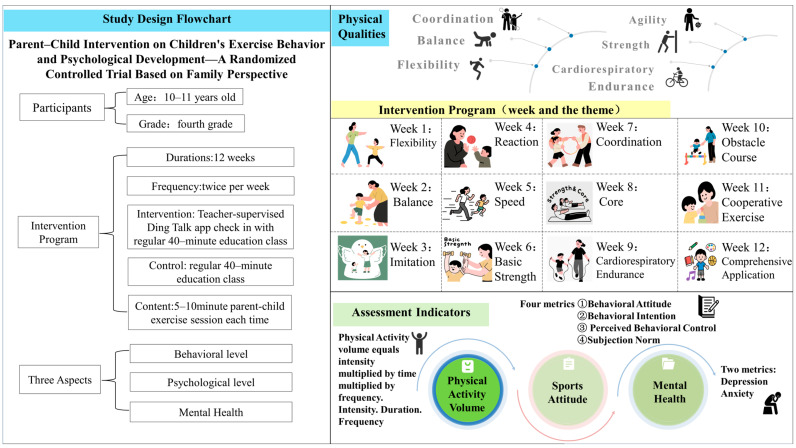
Study Design Flowchart.

Based on the above rationale, this study developed and implemented a standardized 12-week parent–child exercise intervention program (Figure 2).

Intervention Group: In addition to receiving one regular 40-min physical education class per week, the intervention group received two researcher-developed parent–child exercise posts each week through the school’s official channels and digital platforms (Ding Talk APP, developed by Alibaba Group, Hangzhou, China). To ensure the correctness of the exercise movements, the physical education teacher provided brief instruction and demonstration of the core movements for the week’s parent–child exercise tasks before the posts were delivered, helping students grasp the key movement techniques in advance. The posts included gamified parent–child collaborative tasks along with demonstration videos, aimed at guiding guardians and children to complete approximately 5–10 min of structured exercise each time. Participants were required to upload a video of the joint parent–child exercise session via the Ding Talk APP as proof of completion (the video needed to clearly show both parties during the exercise, serving as evidence of actual participation).

To ensure the fidelity and feasibility of the intervention implementation, this study established clear operational procedures and support mechanisms:

(1) Guardian Guidance and Comprehension Assurance Mechanism: Each weekly task package was accompanied by detailed graphic instructions and specially recorded movement demonstration videos. Prior to the official release of the tasks to families, the class PE teacher provided brief explanations and demonstrations of the core movements to students during regular PE classes, enabling students to assist their guardians at home. If the teacher identified common movement errors during video reviews, collective guidance would be delivered in subsequent PE classes. In addition, families could raise questions via the designated platform, thus forming a closed loop of “task delivery → pre-instruction → implementation → feedback”.
(2) Digital Check-in and Verification Process: Families were required to submit a short video (approximately 30–90 s) that clearly showed both parent and child exercising together as check-in evidence within 48 h of task release. The class PE teacher reviewed all videos against criteria, including simultaneous presence of parent and child, correspondence of activity content with the task, and reasonable correctness of movement execution. Reminders were sent for missed or non-compliant check-ins.
(3) Equipment and Resource Requirements: All tasks were designed to require no specialized sports equipment, focusing on bodyweight exercises, imitation, and cooperation. Some tasks suggested using common household items like plastic water bottles or pillows to enhance engagement. No specialized equipment was provided to families, minimizing participation barriers.

To ensure consistency in intervention delivery and the effectiveness of home–school collaboration, the class physical education teacher was responsible for supervising and managing the Ding Talk platform: the teacher reviewed each uploaded exercise video to confirm whether the parent–child pair completed the assigned task as required and whether the movements were performed correctly. Families who failed to check in on time or whose videos did not meet the requirements received gentle reminders and targeted guidance. The teacher also compiled weekly check-in data and reported it to the researchers. Furthermore, the teacher could address questions from students and parents regarding movement techniques during subsequent regular physical education classes, forming a home–school linkage mechanism of “offline pre-guidance, online delivery, video check-in, and offline Q&A.” The entire 12–week program followed the principle of progressive overload and was organized thematically to develop children’s core physical qualities, such as flexibility, balance, reaction, strength, and endurance, constructing a three-stage closed-loop curriculum system: “foundational activation → progressive strengthening → integrated application”. The detailed weekly intervention themes and contents are presented in Table 1.

The weekly parent–child tasks were explicitly designed to incorporate clear goals and progressive challenges to motivate participation and foster a sense of competence. Each task featured specific, achievable performance goals, such as completing a set number of repetitions (e.g., “10 partnered squats”), holding a pose for a target duration (e.g., “a 30-s plank”), or successfully accomplishing a skill-based objective within a limited number of attempts (e.g., “catch the object correctly in 3 tries”). Furthermore, the overall 12-week program was structured to provide progressive challenge across three phases: the foundational phase (Weeks 1–4) focused on mastering basic movements; the developmental phase (Weeks 5–8) introduced greater complexity and coordination demands; and the application phase (Weeks 9–12) presented integrated, game-like scenarios. This structure, based on the principle of progressive overload, ensured that the difficulty level evolved to match the children’s growing capabilities, thereby aiming to sustain engagement and promote skill development throughout the intervention.

Control Group: Participants were only required to attend two regular 40-min physical education classes per week. They did not receive any push materials related to parent–child exercise or additional tasks through any digital platform, serving as the control baseline.

### 2.5. Measures

The outcome measures of this study included physical activity level, mental health, and physical exercise attitude, in order to comprehensively evaluate the intervention effects.

#### 2.5.1. Physical Activity Volume

Subjective assessment was conducted through children’s self-completion of the Physical Activity Rating Scale (PARS-3) (Liang, 1994). This scale, revised by Deqing Liang et al., assesses physical activity volume based on three dimensions: intensity, time, and frequency of physical activity. Physical activity volume score = Intensity score × Time score × Frequency score, with each of the three dimensions scored from 1 to 5 based on a 1–5 scale. This scale has been widely used in Chinese children and adolescent populations (L. Li et al., 2025; P. Wang et al., 2025; K. Zhang et al., 2025). Validation studies within Chinese samples have supported its structural validity, with the three-factor model adequately representing exercise behavior. It has shown moderate correlations with other physical activity measures, supporting its applicability for children in this age group. The reliability of this scale in the current study was satisfactory across all measurement points (see Table 2).

#### 2.5.2. Mental Health

Established scales were used for measurement. The Spence Children’s Anxiety Scale (SCAS) was used to assess anxiety symptoms; this scale was developed by Australian psychologist Susan H. Spence in 1998. This study employed the Chinese short version of the Spence Children’s Anxiety Scale (SCAS-S) (R. Liu et al., 2022) (containing 19 items), covering dimensions such as separation anxiety, social phobia, panic disorder, fear of physical injury, and generalized anxiety. Each item uses a 5-point Likert scale (1 = Never, 2 = Seldom, 3 = Sometimes, 4 = Often, 5 = Always). The Chinese short version has demonstrated good psychometric properties in Chinese children and adolescents (R. Liu et al., 2022). Confirmatory factor analysis supports its five-factor structure, and it shows good convergent and discriminant validity, making it appropriate for anxiety assessment in the current age range.

The Patient Health Questionnaire-9 (PHQ-9) (Kroenke et al., 2001) was used to assess depression levels; this scale is a depression symptom screening and assessment tool developed by American psychiatrist Kroenke et al. Based on the respondent’s actual situation over the past two weeks, it can indicate whether there is a tendency for depression. Each item uses a 4-point Likert scale (1 = Not at all, 2 = Several days, 3 = More than half the days, 4 = Nearly every day). The PHQ-9 has been validated in Chinese adolescent populations, showing a stable unidimensional structure and good sensitivity and specificity for identifying depressive symptoms (Wei et al., 2023), supporting its use in the current study context. Both scales demonstrated high reliability in this study (see Table 2).

#### 2.5.3. Physical Exercise Attitude

The Exercise Attitude Scale compiled by Rongjian Mao (Mao, 2003) was used, and based on Ajzen’s (1991) “Theory of Planned Behavior,” four dimensions were selected for assessment: behavioral attitude, behavioral intention, perceived behavioral control, and subjective norm (31 items in total). Each item uses a 5-point Likert scale (1 = Strongly disagree, 2 = Disagree, 3 = Uncertain, 4 = Agree, 5 = Strongly agree). The scale was developed and validated within a Chinese student population. Its construction was grounded in the Theory of Planned Behavior, and it has demonstrated good construct and criterion validity in subsequent studies with Chinese children and adolescents (Mao, 2003), confirming the stability of its four-factor structure and its relevance in predicting exercise-related outcomes. The reliability coefficients for all subscales were excellent throughout the study (see Table 2).

#### 2.5.4. Questionnaire Reliability and Validity

A pilot survey was conducted on 11 March 2025. Analysis of the 62 valid questionnaires collected indicated that all scales demonstrated acceptable preliminary psychometric properties. In this study, the internal consistency reliability of all multi-item scales was assessed using Cronbach’s alpha coefficient. A Cronbach’s alpha value of 0.70 or above was considered acceptable. To evaluate whether the data from the scales were suitable for examining their factor structure, the Kaiser–Meyer–Olkin (KMO) measure of sampling adequacy was also computed. A KMO value greater than 0.60 is generally considered adequate for factor analysis.

This study collected data from a total of 218 participants. Among them, 112 participants who completed the entire intervention process—including all four measurement points (pre-intervention T1, mid-intervention T2, post-intervention T3, and a 2-month follow-up period)—were included in the final formal analysis. Based on the data from these 112 participants, all scales demonstrated good and stable internal consistency reliability across the four formal measurements, as indicated by Cronbach’s alpha coefficients consistently above 0.6. The KMO values also indicated that the data were suitable for structural evaluation. The detailed results are presented in Table 2.

### 2.6. Statistical Methods

#### 2.6.1. Data Preparation and Normality Tests

This study used SPSS 27.0 software for data analysis. First, descriptive statistical analysis was performed on each continuous outcome variable at baseline, including the calculation of mean, standard deviation, skewness, and kurtosis. Subsequently, the Shapiro–Wilk and Kolmogorov–Smirnov tests were employed to formally assess whether the data met the assumption of normal distribution for parametric tests. The results of these descriptive statistics and normality tests are presented in Table 3.

Normality test results showed that although the K-S test results for all variables were significant (*p* < 0.05), the absolute values of skewness and kurtosis for each variable were below the threshold of 3. This pattern indicates that, while the data deviated from a strictly normal distribution, the deviation was not severe. Therefore, the data were considered to approximate a normal distribution sufficiently to support the use of subsequent parametric statistical analyses, specifically Repeated Measures Analysis of Variance.

#### 2.6.2. Repeated Measures Analysis of Variance

The primary analysis to test the intervention effects on the continuous outcome variables (physical activity volume, exercise attitude dimensions, depression, and anxiety) across the four time points (pre-intervention T1, mid-intervention T2, post-intervention T3, and the 2-month follow-up) was performed using Repeated Measures Analysis of Variance (Repeated Measures ANOVA). This method effectively analyzes changes in measurements for the same subjects at different time points, thereby testing whether the effects of the intervention reached statistical significance. The significance level (α) was set at 0.05. This study interpreted effect sizes according to Cohen’s (1988) guidelines: partial eta squared (ηp^2^) ≥ 0.14 indicates a large effect, 0.06 ≤ ηp^2^ < 0.14 represents a medium effect, and 0.01 ≤ ηp^2^ < 0.06 signifies a small effect (Cohen, 1988).

## 3. Results

### 3.1. Impact of the Intervention on Children’s Physical Activity Volume

**Figure 3:**
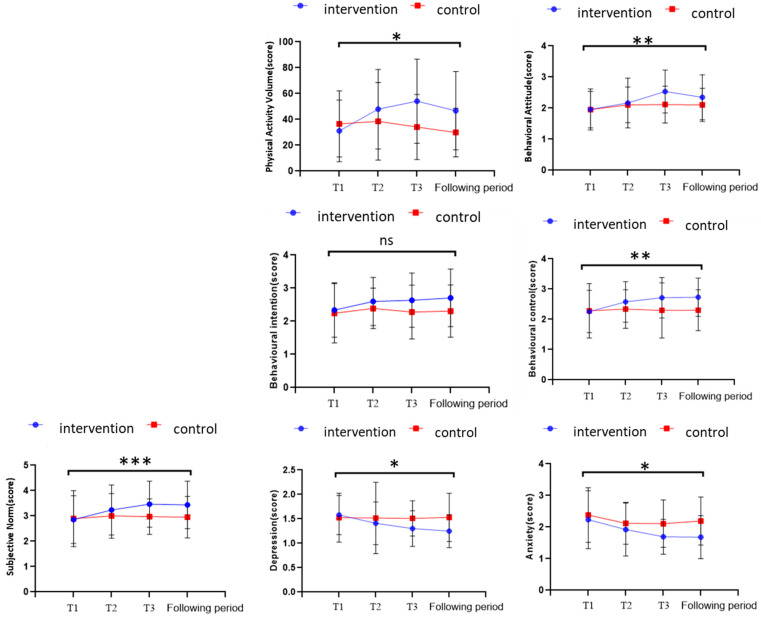
Comparison of Variable Change Trends Between the Two Groups of Children Across Stages. Note: * indicates *p* < 0.05, ** indicates *p* < 0.01, *** indicates *p* < 0.001, ns indicates not significant.

Descriptive data (Table 4 and Figure 3) indicated that children in the intervention group showed an initial increase in physical activity volume during the intervention, which subsequently decreased slightly at follow-up but remained above baseline. In contrast, the control group’s activity levels exhibited only minor fluctuations without a clear trend.

The repeated measures ANOVA (Table 5) revealed a significant group × time interaction effect on physical activity level (F = 17.651, *p* < 0.05, ηp^2^ = 0.138), representing a moderate effect size. Significant main effects were also found for group (F = 4.314, *p* < 0.05) and time (F = 12.697, *p* < 0.005). These results suggest that the intervention effectively enhanced children’s physical activity volume relative to the control condition.

### 3.2. Impact of the Intervention on Children’s Physical Exercise Attitudes

The repeated measures ANOVA results (Table 5) indicated significant group × time interaction effects for behavioral attitude (F = 3.699, *p* < 0.01, ηp^2^ = 0.033), perceived behavioral control (F = 4.189, *p* < 0.01, ηp^2^ = 0.037), and subjective norm (F = 4.616, *p* < 0.001, ηp^2^ = 0.040), though all effect sizes were small. For behavioral intention, while significant main effects were observed for group and time, the interaction effect was not significant (F = 1.346, *p* > 0.05, ηp^2^ = 0.012). This pattern indicates that the intervention positively influenced children’s cognitive and social perceptions toward exercise, but had a less distinct impact on their explicit behavioral planning.

### 3.3. Impact of the Intervention on Children’s Psychological Well-Being

Descriptive statistics (Table 4 and Figure 3) showed that depression and anxiety scores in the intervention group decreased consistently throughout and after the intervention period, whereas the control group exhibited little change.

The repeated measures ANOVA (Table 5) confirmed significant group × time interactions for both depression (F = 4.009, *p* < 0.05, ηp^2^ = 0.044) and anxiety (F = 3.100, *p* < 0.05, ηp^2^ = 0.020), with small effect sizes. Significant main effects for group and time were also present. These findings indicate that the digital parent–child exercise intervention contributed to reductions in children’s depressive and anxiety symptoms over time.

## 4. Discussion

The results of this study indicate that the parent–child intervention produced a medium-sized positive effect on improving children’s physical activity volume. After the 12-week intervention, the intervention group demonstrated a significant increase in mean scores for physical activity volume, showing more substantial advantages than the control group in extending exercise duration, enhancing exercise intensity, and increasing exercise frequency. The parent–child intervention significantly improved children’s physical activity volume through direct parental involvement and support. Emotional support and encouragement from parents boosted children’s self-confidence in facing challenges (Brennan et al., 2025), making them more willing to persist with high-intensity exercise (Gao et al., 2025). Regular parent–child exercise sessions helped children gradually adapt to and develop habits of high-intensity exercise, establishing a consistent routine. As the exercise continued, children’s physical adaptability improved, with enhanced muscle strength, endurance, and cardiorespiratory function (Kolb et al., 2025), enabling them to tolerate higher levels of exercise intensity (Dong et al., 2022). Additionally, integrating physical activity into family life through parent–child interventions, increasing parental support for children’s physical activity, and potentially enhancing parents’ own physical activity volume may all serve as effective approaches to increase children’s physical activity (Saunders et al., 2024). Home–school collaboration further facilitated the extension and reinforcement of motor skills learned at school within the family setting (He, 2022), making the effects of high-intensity exercise more pronounced. The parent–child intervention produced a medium-sized effect on children’s physical activity volume over the 12-week period. This practical meaningfulness can be attributed, in part, to the intervention’s structured design, which embedded explicit performance goals (e.g., repetition counts, time holds) and progressive challenges into the weekly parent–child tasks, all within a supportive home–school collaborative framework. The extent to which this increase can be sustained beyond the intervention period remains a question for future research.

The results of this study also show that after the 12-week intervention, the intervention group exhibited significant improvements in mean scores for behavioral attitude, perceived behavioral control, and subjective norm, demonstrating a more pronounced advantage in physical exercise attitude compared to the control group, although the overall intervention effects were small. First, through active parental involvement and encouragement, especially in play-based parent–child interactions, parent–child interventions can make physical activity more appealing and valuable (Wei et al., 2025), thereby influencing children’s attitude toward exercise—that is, their cognitive evaluation and affective disposition toward physical exercise. Secondly, parental accompaniment and guidance enhance children’s sense of behavioral control, enabling them to believe in their ability to overcome difficulties, which helps sustain their participation in physical activity (Streight et al., 2024). Third, social support and expectation management within parent–child interactions reinforced children’s subjective norm, i.e., their perception of social norms and expectations regarding exercise behavior (F. Wang, 2023). However, these positive changes did not translate into sufficient effect sizes, indicating that the intervention’s impact at the attitudinal level remains limited. Overall, the intervention yielded small but significant improvements in several dimensions of exercise attitude, suggesting a positive preliminary shift in children’s cognitions. However, the relatively weak effect sizes and the standardized task design, which may have limited autonomy, indicate that the current intervention format might be insufficient to foster a strong and stable psychological foundation for long-term adherence. Future adaptations could focus on enhancing personal relevance and choice.

The findings of this study further reveal that after the 12-week intervention, the intervention group showed a significant reduction in mean scores for the two major mental health indicators—depression and anxiety—demonstrating a more substantial advantage in mental health compared to the control group, although the overall intervention effects were small. This indicates that the digital parent–child intervention, through shared participation in physical activity, produced preliminary positive effects on children’s mental health. Parental companionship and interaction provided emotional support and a sense of security, helping alleviate children’s psychological stress and reduce depressive moods (Jin, 2020). Physical activity itself promotes the release of endorphins and other “happiness hormones,” naturally alleviating anxiety and improving mood (Hu et al., 2020). Moreover, successful experiences during shared parent–child exercise and positive feedback from parents enhanced children’s self-confidence and self-efficacy (Ge et al., 2022), further reducing anxiety levels. Through exercise, children also learned strategies to cope with challenges and stress, improving their psychological resilience (Y. Zhao, 2022). The intervention involving both family and school not only strengthened the support network for children’s physical education but also fostered better parent–child communication and understanding, creating a more harmonious and supportive family environment for children (L. Zhao et al., 2023).

In summary, the present study demonstrates that the 12-week digital parent–child exercise intervention exerts positive effects on children’s physical activity volume, exercise attitudes, and mental health. When interpreting the magnitude of the aforementioned intervention effects, it is necessary to conduct a comprehensive analysis in combination with the intervention dosage design of this study. The intervention protocol adopted in this study consisted of parent–child exercise tasks implemented twice a week, with each session lasting 5–10 min. Although the duration of a single session was relatively short, this dosage design was formulated based on comprehensive consideration of behavior change theories, practical feasibility, and dose accumulation effects. First, this intervention was positioned as a structured supplement rather than a replacement for children’s regular in-school physical education classes. Its primary objective was to systematically establish exercise habits and positive parent–child interaction patterns in the family setting through low-threshold, high-frequency interactions, where feasibility serves as the foundation for achieving long-term participation. Second, existing evidence has indicated that regular short-duration, moderate-intensity physical activity yields cumulative health benefits. More importantly, this protocol emphasized transforming children’s attitudes toward exercise, perceived behavioral control, and emotional connection at the social-cognitive level through repeated and successful parent–child interaction experiences. The emotional support, role-modeling, and interactive enjoyment provided by parental participation may have a more crucial impact on psychological outcomes than the mere duration of exercise. Therefore, the observed moderate effects may reflect the synergistic benefits of combining high-quality social interaction with physical activity, rather than being solely attributed to exercise duration. Of course, the dosage design of this study represents a trade-off between optimal effectiveness and practical constraints. Future research could further explore the dose–response relationship of parent–child exercise by setting up groups with varying durations and frequencies, so as to optimize the intervention protocol.

In conclusion, the small but significant reductions in anxiety and depression symptoms provide preliminary evidence that this digital parent–child exercise intervention could be a promising component of strategies aimed at supporting children’s mental well-being. However, these findings must be interpreted with caution due to the short intervention duration, reliance on self-report measures, and the modest effect sizes. The sustainability and clinical relevance of these benefits need robust verification in longer-term trials with more diverse assessment methods.

## 5. Limitations

First, physical activity level was assessed using the Physical Activity Rating Scale (PARS-3) based on children’s self-reports. Although this scale is widely used and offers high practicality, it is a subjective measure and thus susceptible to social desirability bias (e.g., children in the intervention group may overreport their activity, intentionally or unintentionally) and recall bias. This may introduce some error in estimating the intervention effects, particularly on physical activity volume. While we implemented assessor blinding and anonymous data analysis to mitigate some bias, future studies incorporating objective measures such as accelerometers or heart rate monitors would provide a more accurate and reliable assessment of physical activity frequency, intensity, and duration, thereby verifying and extending the findings of this study.

Second, although the digital parent–child intervention in this study achieved significant success in enhancing children’s physical activity volume, behavioral attitude, perceived behavioral control, and subjective norm, no significant difference was observed between the intervention and control groups in the dimension of behavioral intention (F = 1.346, *p* > 0.05). This finding suggests that the mechanisms through which the parent–child intervention influences children’s formation of “I am willing” and “I plan to” exercise intentions may be more complex. First, regarding intervention design, the standardized push notification model adopted to ensure implementation efficiency may have somewhat limited the satisfaction of children’s need for autonomy, thereby affecting the internalization of intention. According to Self-Determination Theory, an individual’s need for autonomy—feeling that behavior is voluntary and self-chosen—is central to stimulating high-quality motivation and stable behavioral intention (F. Wang, 2023). The uniform content and fixed procedures in this study inadvertently transformed parent–child exercise into a mandatory “task.” When activities lack room for personal choice, children’s motivation can easily shift from “I want to do it” to “I have to do it.” Participation driven primarily by such external regulation struggles to be efficiently translated into strong and enduring autonomous behavioral intention. Second, regarding task design, insufficient sustained task appeal and challenge may have undermined the accumulation of a sense of competence. Another key element of Self-Determination Theory is competence, the feeling of being effective in an activity (Yang & Zhou, 2025). Although the intervention program was richly designed, the difficulty of the pushed tasks was pre-set and uniform, failing to match the dynamically developing skill levels of all children. For some children, tasks that are consistently too simple can lead to boredom, while those that are occasionally too difficult can cause frustration. Neither scenario provides continuous and appropriate challenge, thus hindering children from gaining a sufficient sense of competence from the activities. A lack of competence directly weakens the intrinsic willingness and intention to continue participating.

Furthermore, the primary statistical analysis (Repeated Measures ANOVA) did not include baseline scores or other potential covariates (e.g., family socioeconomic status) as covariates in the model. Our analysis relied primarily on randomized group allocation and the comparability of groups on all outcome measures at baseline to estimate intervention effects. While this approach is consistent with the basic logic of the design and allows for a direct test of the group-by-time interaction, future studies could provide a more precise estimate of the net intervention effect by employing models such as Repeated Measures ANCOVA, which control for baseline values and potentially other relevant covariates.

Based on these limitations, future research could explore more personalized and adaptive intervention programs. For instance, while maintaining the core framework, families could be allowed a certain degree of autonomy in choosing activity types or difficulty levels to better satisfy children’s need for autonomy. Simultaneously, preliminary ability assessment mechanisms could be introduced to dynamically adjust task challenge based on children’s real-time feedback and performance, ensuring tasks remain within their “zone of proximal development,” thereby more effectively fostering competence formation and stimulating behavioral intention.

## 6. Conclusions

This 12-week randomized controlled trial preliminarily investigated the multi-dimensional effects of a digital platform (Ding Talk)-based, home–school collaborative parent–child exercise intervention on children’s health. The main findings are summarized as follows: First, the intervention significantly increased children’s physical activity volume, with a medium effect size. This result demonstrates the feasibility of using structured, gamified parent–child tasks combined with a closed-loop home–school management system (featuring teacher pre-guidance, online check-in, and feedback) to effectively promote moderate-to-vigorous physical activity among children in the home setting. Second, the intervention produced statistically significant, albeit small, positive effects on children’s exercise attitudes and mental health. Specifically, positive trends were observed in children’s behavioral attitude towards exercise, perceived behavioral control, and subjective norm; concurrently, a slight reduction in symptoms of depression and anxiety was noted. These findings provide preliminary empirical support for the notion that joint parent–child participation in physical activity may benefit children’s psychosocial development.

In conclusion, this study supports integrating low-barrier, easily implementable digital parent–child exercise as a potentially valuable health-promotion module into existing school-family collaborative systems. It offers a practical reference model for implementing feasible child health interventions in real-world settings. Future research should focus on optimizing the intervention protocol (e.g., exploring personalized tasks, increasing autonomy), extending the intervention and follow-up periods, and examining its long-term efficacy and mechanisms within broader populations. This will lay a more solid scientific foundation for developing more impactful comprehensive health promotion strategies for children.

## Figures and Tables

**Table 1:** Detailed Parent–Child Exercise Intervention Schedule.

Intervention Week	Theme	Content
1	Flexibility	1. Flexibility Magic Workout: Sumo Stretch; Lunge Stretch; Side Split Stretch; Gorilla Walk; Crawling Lizard 2. Parent–Child Fun Flexibility Games: Little Horse Stretch; Wheel Roll; Pass Supplies; Pass Water; Capture the City.
2	Balance	1. Static & Dynamic Balance Routine: Single Leg Front Lift; Single Leg Side Lift; Single Leg Back Lift; Hip Extension Hold; Single Leg Front Kick 2. Family Balance Fun Show: Balance Fitness Training 1; Balance Fitness Training 2; Balance Fitness Activity 1; Balance Fitness Activity 2.
3	Imitation	1. Imitation Challenge: Mirror Reflection; Parent–Child Imitation Show; Symmetrical Grab Game 2. Imitation Training Games: Family Copy Machine; “1,2,3” Statue; Parent–Child Mirror Simulation; Joint Transport.
4	Reaction	1. Flash Adventure: Horizontal Melee; Number Bomb; Reaction Master 2. Lightning Reaction Challenge: Rhythm Master; You Throw I Catch; Pinwheel.
5	Speed	1. Speed Competition: Circle Chase; High Toss Quick Catch; Music Shuttle Run 2. Swift as the Wind: Octopus Sprint; Judgment Expert; Judgment Expert Advanced Challenge.
6	Basic Strength	1. Basic Strength Face-off: Bottle Tug-of-War; Partner Jump; Plank Competition; Plank Competition Advanced 2. Parent–Child Basic Strength Training: Mutual Assistance; Push and Shove; Relay High-Five; Lunge Step Cross; Squat Leg Lift; Waist Twists Left-Right Front-Back.
7	Coordination	1. Parent–Child Coordination Workout: Agile Catch & Throw Challenge 1; Agile Catch & Throw Challenge 2; Rope Catch & Throw Position Swap 2. Coordination Rhythm Competition: Ball Bounce Touch Relay Show 1; Hand–Foot Cross Speed Challenge 1; Hand–Foot Cross Speed Challenge 2; Hand–Foot Cross Speed Challenge 3; Hand–Foot Cross Speed Challenge 4.
8	Core	1. Conquer the Core Domain: Hovering Catch Challenge; Quadruped Guardian; Knee-to-Knee Twist Code; Hoop Body Passer; Touch Ground Turnaround Battle 2. Seize the Core Power: Hovering Foot Control Master; Palm Sprint Relay; Air Clamp Transport Expert; Side Dodge Mutual Warrior; Center of Gravity Control Master.
9	Cardiorespiratory Endurance	1. Cardio Endurance Cooperation Pioneers: Burpee Squat Jump; Flip Paper Reversal; The Fool Moves the Mountain 2. Cardio Overcoming Challenges: Leg Sweep; Parent–Child Ball Catch; GO! GO! Operation
10	Obstacle Course	1. Puzzle Mission: Rock-Paper-Scissors High Jump; Walk with Shoes; Manipulate the Puppet 2. Unified Hearts Knot 1 + 1=11: Climb Mountains and Cross Ridges; Steady as a Mountain; Touch Ground Storm.
11	Cooperative Exercise	1. Unified Hearts and Shared Journey, Collective Victory Single Foot Moves the Universe; Bottle Hit Time Trial; Prone Unified Force 2. Progress Together, Energy as One Leaping Catch & Hit Competition; Winding Path of Unity; Agile Threading the Clouds Pass.
12	Comprehensive Application	1. Role-Playing Carnival: Small Ball Crosses the River; Blow Up the Enemy Camp; Human Obstacle Run 2. Living Room Olympics: Grabbing Hands Competition; Veteran Cross-Country Running; Step on the Tail.

**Table 2:** Internal Consistency (Cronbach’s α) and Sampling Adequacy (KMO) of Scales at Each Stage.

Variable	T1	T2	T3	Follow-Up Period
	α (KMO)	α (KMO)	α (KMO)	α (KMO)
Physical Activity Volume	0.596 (0.591)	0.636 (0.549)	0.561 (0.520)	0.641 (0.570)
Behavioral Attitude	0.689 (0.854)	0.772 (0.918)	0.771 (0.886)	0.740 (0.897)
Behavioral Intention	0.781 (0.741)	0.719 (0.693)	0.806 (0.740)	0.820 (0.758)
Perceived Behavioral Control	0.769 (0.785)	0.764 (0.885)	0.801 (0.886)	0.806 (0.864)
Subjective Norm	0.863 (0.877)	0.918 (0.893)	0.906 (0.900)	0.902 (0.862)
Depression	0.809 (0.787)	0.863 (0.843)	0.820 (0.783)	0.862 (0.855)
Anxiety	0.931 (0.873)	0.917 (0.876)	0.920 (0.873)	0.928 (0.844)

**Table 3:** Normality Test Results for Each Variable’s Data.

Variable	Mean	Standard Deviation	Skewness	Kurtosis	K-S Test		S-W Test	
					D Value	*p* Value	W Value	*p* Value
Physical Activity Volume	41.065	29.071	0.84	0.098	0.129	0.000	0.927	0.000
Behavioral Attitude	2.182	0.691	0.647	0.143	0.09	0.000	0.962	0.000
Behavioral Intention	2.467	0.815	0.277	−0.11	0.077	0.000	0.976	0.000
Perceived Behavioral Control	2.468	0.74	0.097	−0.182	0.065	0.000	0.985	0.000
Subjective Norm	3.136	0.948	−0.057	−0.139	0.093	0.000	0.974	0.000
Depression	1.453	0.485	1.77	2.062	0.175	0.000	0.823	0.000
Anxiety	2.008	0.8	0.963	0.712	0.113	0.000	0.926	0.000

**Table 4:** Descriptive Statistics of Variables for the Two Groups of Children at Different Stages.

Variable	Intervention Group				Control Group			
	Pre	Mid	Pos	Follow-Up	Pre	Mid	Post	Follow-Up
Physical Activity Volume	31.14	47.95	54.15	46.68	36.51	38.58	34.13	29.86
Behavioral Attitude	1.958	2.166	2.533	2.340	1.951	2.105	2.114	2.105
Behavioral Intention	2.337	2.598	2.635	2.703	2.242	2.388	2.279	2.308
Perceived Behavioral Control	2.254	2.579	2.715	2.731	2.281	2.337	2.293	2.302
Subjective Norm	2.856	3.235	3.463	3.436	2.895	3.000	2.971	2.953
Depression	1.561	1.444	1.347	1.268	1.534	1.529	1.529	1.519
Anxiety	2.235	1.919	1.721	1.708	2.406	2.068	2.097	2.176

**Table 5:** Results of Repeated Measures Analysis of Variance for Children’s Variables.

Variable	F-Values			Partial Eta Squared (η^2^)		
	Interaction	Between-Group	Within-Group	Interaction	Between-Group	Within-Group
Physical Activity Volume	17.651 *	4.314 *	12.697 ***	0.138	0.038	0.103
Behavioral Attitude	3.699 **	3.4	10.385 ***	0.033	0.030	0.086
Behavioral Intention	1.346	5.094 *	2.826 *	0.012	0.044	0.025
Perceived Behavioral Control	4.189 **	6.314 *	4.857 ***	0.037	0.054	0.042
Subjective Norm	4.616 ***	3.903 *	7.145 ***	0.040	0.034	0.061
Depression	4.009 *	5.225 **	5.093 **	0.044	0.035	0.045
Anxiety	3.1 *	7.006 ***	12.617 ***	0.020	0.051	0.096

Note: * indicates *p* < 0.05, ** indicates *p* < 0.01, *** indicates *p* < 0.001.

## Data Availability

The datasets used and/or analyzed during the current study are available from the corresponding authors upon reasonable request.
